# Chrysalis and Butterfly

**Published:** 1893-06

**Authors:** 


### EDITORIAL DEPARTMENT.

F. E. DANIEL, M. D., Editor.
S. E. HUDSON, M. D., Managing Editor.
A. J. SMITH. M. D., Galveston, Associate Editor.

EDITORIAL STAFF:

PROF. J. E. THOMPSON, M. D., Texas Medical College, Galveston; Surgery.
PROF. WM. KEILLER, M. D., Texas Medical College, Galveston; Obstetrics and Gynecology.
PROF. DAVID CERNA, M. D., Texas Medical College, Galveston; Therapeutics.
PROF. A. J. SMITH, M. D., Texas Medical College, Galveston; Medicine.
DR. ISADORE DYER, Tulane University; Dermatology.
DR. R. H. L. BIBB, Saltillo, Mexico; Foreign Correspondent.
DR. ROBT. MORRIS, Charity Hospital, N. O.; Clinical Reports.

The JOURNAL is the official organ of the Austin District Medical Society, the Wes Texas District Medical Society and the Galveston County Medical Society.

### CHRYSALIS AND BUTTERFLY.

### Salutatory.

SHAKE, BRETHREN! This number of DANIEL'S TEXAS MEDICAL JOURNAL completes Vol. 8. It rounds out and finishes the *eighth year* of its sunny existence,—for its career has been like a summer day, unclouded by trouble—unhindered by any misfortune. It has flourished, thank you, like the fabled and famous green bay-tree; prospered and grown like a well fed and healthy infant; grown and grown, till, in fact,—it has gotten too big for its breeches, (figuratively speaking and in a Pickwickian sense). Up, up, up, higher and higher has it climbed the ladder of success till it has left all competitors, and is, itself, in the vernacular of the day, *away out of sight*. (How is that for high?) So, this JUNE number is a *memorial* number; it commemorates at once, the *birthday*, and the *demise* of DANIEL'S TEXAS MEDICAL JOURNAL. Nay, start not; not dead,—nor sleepeth, not by a large majority. It has simply changed form,—cast off its too small trousers, —nickerbockers, so to, speak, and with *July* will don the long breeches of the full grown man!

*That is to say*,—it is no longer *Daniel's Texas Medical Journal;* but

THE TEXAS MEDICAL JOURNAL!

(Daniel & Hudson, owners, editors and proprietors.) That's funny, you may say; a distinction without a difference. Not quite. Did you ever think about it? The "*Daniel's*" is, in a sense, an adverb, *qualifying*,—telling the *kind* of Texas Medical Journal it is (or was). From which, it might be inferred that there are or might be, *other* Texas Medical Journals,—clearly a mistake;—for instance,—one could infer, if *this* is *Daniel's* Texas Medical Journal, *Smith* may, can, must, might, could, would, or should have, somewhere, a *Texas* Medical Journal. It is like the devoted young wife, who when she received a letter from "Hubby" addressed to his "dearest Maria," sensible (and jealous) woman as she was, "burst into tears" [used by permission, copy righted by Ned Buntlin, I believe,] and exclaimed, "where, Oh where are his *other* dear Marias! if I am his dearest Maria? show me to them; do."

*Which is to say*—there is but *one* TEXAS MEDICAL JOURNAL and this is the identical! It fills the requirements; covers the ground; and, to tell the truth, there is *no room*, even in this great big State for any other *Texas Medical Journal.*

As we make our *conge* after the 8th act, we make our bow and beg to be excused while we go and change our——*knickerbockers.*

Our July number will be a beauty. She will sail out under the title "TEXAS MEDICAL JOURNAL;" and we announce now, and wish it to be borne in the mind that under *this* "signo," we will continue to "vinces" as in the past we "vince"d under the original title.

With cordial thanks to all our friends who have contributed so liberally to the success of *Daniel's Texas Medical Journal* we ask of the Texas profession a warm and generous support in our earnest endeavors to give them and the State the *best medical journals published any where!*

